# Multimodal SARS-CoV-2 interactome sketches the virus-host spatial organization

**DOI:** 10.1038/s42003-025-07933-z

**Published:** 2025-03-26

**Authors:** Guillaume Dugied, Estelle MN Laurent, Mikaël Attia, Jean-Pascal Gimeno, Kamel Bachiri, Payman Samavarchi-Tehrani, Flora Donati, Yannis Rahou, Sandie Munier, Faustine Amara, Mélanie Dos Santos, Nicolas Soler, Stevenn Volant, Natalia Pietrosemoli, Anne-Claude Gingras, Georgios A. Pavlopoulos, Sylvie van der Werf, Pascal Falter-Braun, Patrick Aloy, Yves Jacob, Anastassia Komarova, Yorgos Sofianatos, Etienne Coyaud, Caroline Demeret

**Affiliations:** 1 Institut Pasteur, Université Paris Cité, UMR 3569, Centre National de la Recherche Scientifique, Molecular Genetics of RNA Viruses, 28 rue du Docteur Roux, F-75015 Paris, France; 2 Univ. Lille, Inserm, CHU Lille, U1192 - Protéomique Réponse Inflammatoire Spectrométrie de Masse - PRISM, F-59000 Lille, France; 3 Lunenfeld-Tanenbaum Research Institute at Mount Sinai Hospital, Sinai Health, Toronto, ON Canada; 4 Institut Pasteur, Université Paris Cité, National Reference Center for respiratory viruses, 28 rue du Docteur Roux, F-75015 Paris, France; 5 Institute for Research in Biomedicine (IRB Barcelona), The Barcelona Institute of Science and Technology, Baldiri Reixac 10 -12, 08020 Barcelona, Spain; 6 Bioinformatics and Biostatistics Hub, Institut Pasteur, Université Paris Cité, F-75015 Paris, France; 7 Institute for Fundamental Biomedical Research, BSRC “Alexander Fleming”, 34 Fleming Street, 16672 Vari, Greece; 8 Institute of Network Biology (INET), Molecular Targets and Therapeutics Center (MTTC), Helmholtz Center Munich, German Research Center for Environmental Health, Munich-Neuherberg, Munich, Germany; 9 Microbe-Host Interactions, Faculty of Biology, Ludwig-Maximilians-Universität (LMU) München, Planegg-Martinsried, Munich, Germany; 10 Institució Catalana de Recerca i Estudis Avançat (ICREA), Pg. Lluís Companys, 23, 08010 Barcelona, Spain; 11 Present Address: Institut Pasteur, Université Paris Cité, Interactomics, RNA and Immunity, 28 rue du Docteur Roux, F-75015 Paris, France

**Keywords:** SARS-CoV-2, Network topology

## Abstract

An accurate spatial representation of protein-protein interaction networks is needed to achieve a realistic and biologically relevant representation of interactomes. Here, we leveraged the spatial information included in Proximity-Dependent Biotin Identification (BioID) interactomes of SARS-CoV-2 proteins to calculate weighted distances and model the organization of the SARS-CoV-2-human interactome in three dimensions (3D) within a cell-like volume. Cell regions with viral occupancy were highlighted, along with the coordination of viral proteins exploiting the cellular machinery. Profiling physical intra-virus and virus-host contacts enabled us to demonstrate both the accuracy and the predictive value of our 3D map for direct interactions, meaning that proteins in closer proximity tend to interact physically. Several functionally important virus-host complexes were detected, and robust structural models were obtained, opening the way to structure-directed drug discovery screens. This PPI discovery pipeline approach brings us closer to a realistic spatial representation of interactomes, which, when applied to viruses or other pathogens, can provide significant information for infection. Thus, it represents a promising tool for coping with emerging infectious diseases.

## Abstract (web summary)

The modelling of the SARS-CoV-2 spatial organization within a cell-like volume combined with physical virus/host contacts profiling brings a realistic spatial representation of SARS-CoV-2/human interactome.

## Introduction

Proteins are the main agents involved in the regulation of biological functions and don’t act alone to mediate and regulate these functions; instead, they act in concert with other proteins through their interactions^1–5^. These protein-protein interactions (PPIs) are involved in the development of human diseases, either genetic, physiologic or infectious^6–8^. Therefore, mapping PPIs is essential to understanding their role in the development of diseases at the cellular level. Representation of networks of interactions is a critical component of the analysis, allowing the identification of proteins involved in similar complexes or pathways. However, PPI networks are often incomplete and hard to exploit, primarily due to the intrinsic nature of PPI detection methods and the two-dimensional network representations. Additionally, most interaction networks only represent a single study, performed with a single PPI detection method, while a complete network would require the aggregation of multiple studies. Moreover, distances within a protein interaction network are often empirical and without biological meaning, precluding the representation of actual volumes and regions. Contrasting with the huge increase in PPI detection methods sensitivity and deepness, little if any progress has been made toward the spatial representation of PPI networks. The tools developed to extrapolate distances from PPI networks dating back more than 10 years^9,10^. The inference of protein locations from interaction data to generate a spatial representation of interaction networks has so far remained out of reach. This step is yet a prerequisite towards an accurate three-dimensional representation of the PPI network within the cell volume.

Current layouts are often used to highlight network structure in two dimensions (2D) but rarely in three (3D) due to the inherent difficulties in displaying the actual depth information, resorting to just projections on two dimensions. However, especially in larger or dense networks, the flattening distorts the richer 3D structure and inevitably collapses important information. Moreover, proteins reside inside an actual, physical cell volume that is three-dimensional, so any attempt to provide a picture of the spatial organization of their interactions should take that into account. Here, motivated by the fact that our experimental PPI detection methods capture spatial proximity information, we set out to develop a methodology to provide an approximate placement for the proteins in 3D space. Using prior ideas from graph layouts extended to suit our purposes^11–13^, we find that the amount and quality of the proximal interaction data provide enough information to efficiently guide the graph 3D layout towards a picture largely consistent with pre-existing knowledge, as well as other studies, even orthogonal ones. Below, we report on these findings as applied to the SARS-CoV-2 virus-host interactome.

COVID-19, the disease caused by the Severe Acute Respiratory Syndrome Coronavirus 2 (SARS-CoV-2), has been shown to be intricately correlated to SARS-CoV-2-host PPIs, which have been extensively studied using different detection methods^14–20^. However, no studies have aggregated and represented the complete SARS-CoV-2-host interactome. In this study, we present a new methodology to calculate the relative positions of viral and host proteins in an abstract representation of the cellular space using a proximity-dependent biotinylation (BioID) dataset as our starting point. We then modeled the spatial representation of the SARS-CoV-2/human interactome, revealing the invasion pattern of cell territories by the viral proteins and reflecting virus coordination to coopt the cell machinery along the viral cycle. Furthermore, by profiling direct virus-host contacts within the SARS-CoV-2-human proximal interactome, we have confirmed the accuracy of the three-dimensional map and demonstrated its predictive value for direct PPI identification. Functionally important intra-virus and virus-host PPIs have been detected^21–24^, and numerous direct virus-host contacts involving human proteins have contributed to infection. We also provided robust modeled structures of virus-host complexes, paving the way for structure-directed functional exploration or to anti-viral therapeutics development. Our work lays a foundation for the spatial representation of interaction maps using BioID datasets. When supplemented by the comparative profiling of direct virus-hosts contacts, our approach facilitates the generation of more sophisticated and informative interaction map.

## Results

### Modeling the spatial organization of SARS-CoV-2-host proteome from the proximal interactome

**Fig. 1:**
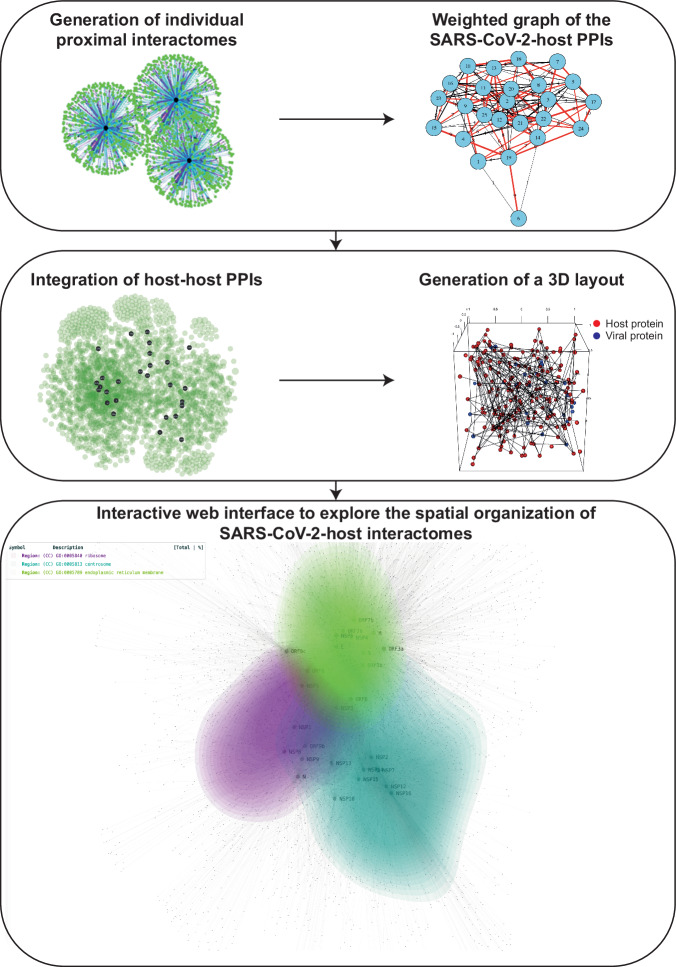
Illustration of the computational pipeline. The BioID virus-host proximal interactomics datasets are combined and encoded into a weighted graph data structure. This structure is augmented with previously reported host-host interactions, and a multi-stage and force-directed graph layout is used to generate 3D coordinates of all nodes. The modeled 3D map can be accessed in a web-based interactive display, with option for with filtering and customization features. Further integration with other available interactomes from a variety of study types resulting in a global comparative interactome view.

**Fig. 2:**
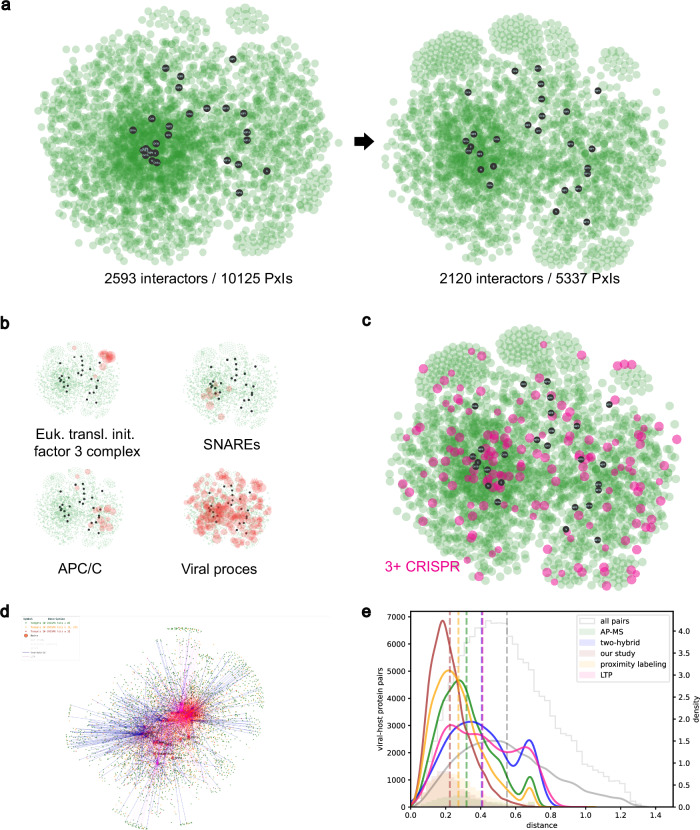
SARS-CoV-2-human proximal network representation. **a** Global data-driven SARS-CoV-2-human proximal network projection on 2D and its relaxation upon removal of the host proteins targeted by eight or more viral baits. PxIs stands for proximal interactions. **b** Spatial localization of functional groups of proteins in the proximal network relative to the viral proteins (black nodes), as indicated. **c** Localization of human proteins essential for SARS-CoV-2 induced toxicity (3 + CRISPR hits) on the SARS-CoV-2-host 2D proximal interaction map. **d** Picture of the global comparative view of the SARS-CoV-2-host interactomes, highlighted in blue, red and pink are the SARS-CoV-2-host interactions identified in two-hybrid, low throughput study and our BioID study, respectively. **e** Histograms and densities of viral-host protein pair distances in the global 3D SARS-CoV-2-host proximal interaction map and breakdown by their origin study type.

Since BioID bait proteins label proximal partners within a volume of ~10–20 nm radius^25^, proximal interactomics data contain spatial information about the proteins’ vicinity. Based on this property, we developed a methodology to approximate the relative position of viral and host proteins inside an abstract representation of cellular space (detailed in the Methods section). We used the SARS-CoV-2-host proximity interaction map of 27 SARS-CoV-2 proteins that we generated by proximity-dependent biotin identification (BioID)^26^ (Supplementary Tables 1 and 2). Comparison of our BioID interactome with several meta-analyses of SARS-CoV-2 interactomes confirmed the quality of our dataset. Indeed, we detected 143/332 (43%) of the PPIs identified in multiple AP-MS studies listed in Hoffmann et al.^27^. We also recovered 125/298 (42%) and 920/1086 (85%) of the highly filtered SARS-CoV-2/host PPI detected by AP-MS or by proximal interactomics respectively and provided in Li et al.^28^. Last, from the list of 11,755 unique SARS-CoV2/host PPIs gathered in Sheng et al.^29^, more than a third (4,051/11,755) were also present in our BioID data. We started by modeling the pairwise BioID proximal interactions from the aggregated individual proximal interactomes^26^ into a weighted graph structure, fully capturing the information of the SARS-CoV-2 proximal interactome. We then introduced additional edges to the graph, corresponding to previously reported host-host protein interactions, which helps refine the connectivity with already-known information about the host proteome. By applying a force-directed graph layout algorithm in three dimensions, we could determine a set of 3D coordinates representing the positions of viral and host proteins. The coordinates allowed us to infer relative distances between nodes within our abstract 3D space (contained within a cube of [−1,1]x[−1,1]x[−1,1] in non-physical arbitrary units), compare proximities and observe general regions of viral interaction (Fig. 1). Integration with other SARS-CoV-2 metadata and orthogonal interactomics studies supported the accuracy of the targeted cellular regions and of the viral proteome organization, confirming the reliability of the 3D modeling. The generated virus-host interaction map is available via an interactive web interface (accessible at http://dev.sars-cov-2-interactome.org/) and can be used to visualize and explore the SARS-CoV-2-host proteins spatial organization through various customization options, such as visualization of the virus/proteome interface based on GO-terms filtering. While not possible to establish a precise geometric correspondence between our generated 3D volume and the cell’s physical volume due to experimental but also graph modeling limitations (e.g. in the case of multiple protein localizations which cannot be accurately captured by a single node location), the map provides an approximation of the spatial organization of viral and host proteins, enabling a sketch of their interplay inside the actual physical cell volume (see Methods). Importantly, the BioID samples from the individual proximal interactomes were prepared in the presence of turbonuclease, a potent DNA and RNA nuclease that cleaves both single stranded and double stranded nucleic acids. We thus expect the proximal interactions detected by this technique to be independent of nucleic acids. The virus-host network layout revealed a cluster of 13 viral proteins (ORF3a, ORF3d, M, S, E, NSP3, NSP4, NSP6, ORF8, ORF7a, ORF7b, ORF6 and ORF9c) concentrated in the same proteome environment, surrounded by 14 viral proteins pulled away by different sets of partners. The viral cluster consists in organelle-associated proteins that all gather around a dense core of host factors, some of which likely present due to their colocalization in the same subcellular compartments. To get rid of as much of such compartment specific background without altering biological insights of the BioID dataset, we assessed how filtering out host factors according the their interaction degree with the viral proteins affects the fraction of proximal interactions per viral factor (Supplementary Fig S1A). We observed that removing from the BioID dataset the most highly connected host proteins primarily reduced the proximal interactions of the core viral proteins, without significantly affecting PPIs detection of the peripheral ones, suggesting that most of the higher degree host proteins represent compartment-specific background. Filtering out host proteins with eight or more viral interaction partners induced the relaxation of the dense core in the 3D map (Fig. 2a, Supplementary Fig. S1A-B). Examining the overall node density of host proteins in our 3D layout, we observe a sharp decrease with radial distance from the cluster’s centroid, whereas by restricting to proteins with a maximum of seven viral interactions, the node density exhibits a peak followed by a smooth decrease with radial distance (Supplementary Fig. S1C). Using the radial value corresponding to the density inflection point to define a threshold for the cluster size (or *dense core*) enabled us to identify a list of 790 host factors residing within the dense core boundary, which are in their vast majority assigned to membrane-associated categories (Supplementary Table 3). Membrane-rich organelles are the known primary locations of nine viral proteins (ORF3a, M, S, E, NSP3, NSP4, NSP6, ORF7a and ORF7b)^30^, all of which are included in the dense core. Expectedly, when removing host proteins with degree 8 or higher, the dense core was relaxed while keeping membrane-bound host proximal partners of a specific set of viral proteins. Hence, this operation facilitates the extraction of discrete proximity-based hypotheses for each viral protein. Regions of pathway-specific subnetworks can also be captured, revealing their positioning relative to the SARS-CoV-2 proteins and highlighting the coordination between viral proteins to exploit host cells (Fig. 2b and Supplementary Table 2). When intersecting our BioID dataset with SARS-CoV-2-related CRISPR-Cas9 studies available at BioGRID ORCS, we identified 187 host factors reported as important for SARS-CoV-2-induced cytotoxicity in ≥3 CRISPR-Cas9 and connected to 27 viral bait proteins through 694 proximal interactions (Fig. 2c and Supplementary Table 2). The map reveals the positioning of functionally relevant host proteins within the proximal landscape of viral polypeptides, which largely follow the overall node radial distribution (Supplementary Fig. S1D). In all, the 3D SARS-CoV-2-host map provides unique insights into the intricate host proteome hijacking and is capable of capturing the spatial distribution of targeted complexes or processes, including essential ones pertaining to the SARS-CoV-2 proteome.

### Modeling the complete spatial SARS-CoV-2-host contactome

Several SARS-CoV-2-host interactomes produced using different methods are available in the literature (references in Supplementary Table 2). We utilized these datasets to obtain a complete picture of the SARS-CoV-2-host interplay identified to date, employing the same modeling and 3D visualization approach to augment the map generated by our own study. The overall shape of the SARS-CoV-2-host 3D map is preserved, indicating that the graph layout approach provides consistent viral-host neighborhoods across SARS-CoV-2 interactomics studies. Each released interactome is made separately accessible in the global SARS-CoV-2-host 3D map, which makes observable the different features of each dataset, such as the expected underrepresentation of membrane-associated PPIs obtained in the AP-MS dataset (“Global comparative view” at http://dev.sars-cov-2-interactome.org/).

We compared the Euclidean distances in our 3D map of the viral-host protein pairs for every PPI detection method for which datasets were available in the literature (references in Supplementary Table 2) (Fig. 2e, Supplementary Table 4). The average distance of virus-host protein pairs from our own study is lower compared to those of any other study (mean of 0.21), which is expected since the 3D map has been created using our study’s proximal dataset. We individually compared the total distribution of the viral-host protein pairs distances of each PPI set of data with the subsets present and absent in our dataset. We observed a bimodal distribution of the viral-host protein distances for data coming from every PPI detection method but ours. We found that the most distant peak observed with each other PPI datasets consists of protein pairs that were not present in our BioID dataset (Supplementary Fig. S1E–H). Of note, the High Confidence viral-host protein pairs identified in ≥2 AP-MS studies or in ≥3 Proximity Labeling studies as in Li et al.^28^, showed an overall median distance of 0.25 and 0.18 respectively, while the whole PPIs matrix shows a median distance of 0.52 (Supplementary Fig. S1I). These data support the robustness of the 3D map to position positive viral-host interactions to closer distances accurately.

Overall, our proximal interactome-based modeling pipeline provides the 3D representation of the most complete SARS-CoV-2-host interactome to date, marking a step towards a more realistic picture of the spatial positioning of SARS-CoV-2 in the cell proteome while at the same time significantly advancing the state-of-the-art in the representation of protein-protein interaction networks in general.

### Exploring physical contacts of the viral proteins in the proximity interactome dataset

**Fig. 3:**
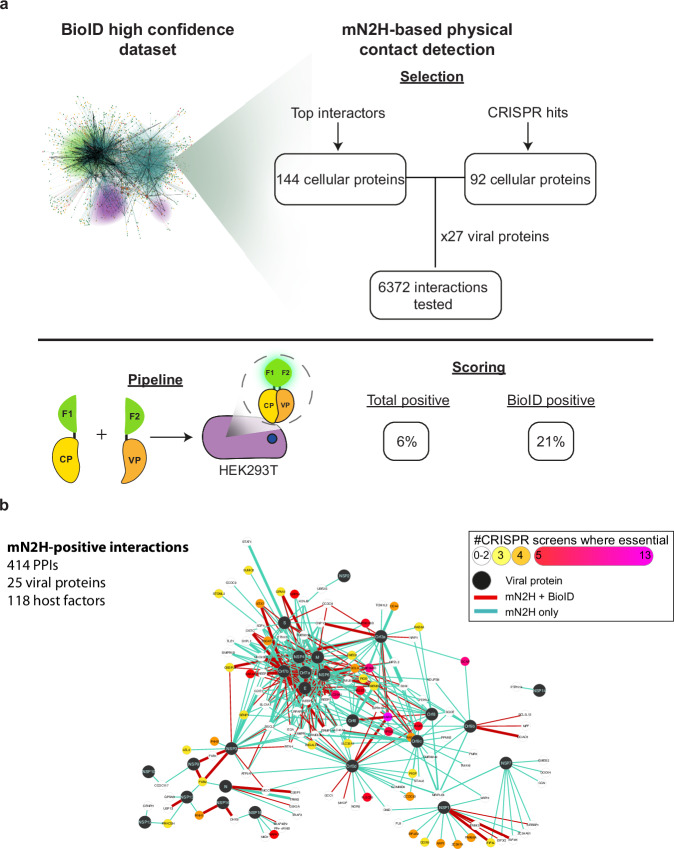
mN2H orthogonal validation of a subset of high confidence interactors. **a** Selection of the interactors and experimental pipeline for the mN2H validation. **b** Full SARS-CoV-2-human contactome network identified in this study by mN2H.

Combining orthogonal PPI detection methods is a well-established approach to increasing both the accuracy and coverage of large-scale PPI maps. We complemented our BioID proximal interactomics dataset with a split-nanoluciferase assay, which senses binary PPIs by protein-fragment complementation of the nanoluciferase enzyme in human cells (mN2H) and offers excellent detection performance^31^. PPI screens by mN2H are systematic (matrix-based) and semi-quantitative (luciferase-based intensity map of interactions), providing a solid orthogonality to BioID (see methods). We sampled two sets of factors from our BioID dataset for mN2H-based PPIs profiling with the SARS-CoV-2 proteins (Fig. 3a) : (**i**) 144 host factors highly enriched with less than four viral bait proteins (based on log2 fold change over background and q-value significance); and (**ii**) 92 host factors scoring positive in 3 + CRISPR-Cas9 screens. The selected host factors were tested against the 27 viral proteins by mN2H, thereby representing a complementary PPI screening which will provide comparative virus-host interaction profiles. The full mN2H matrix interrogated about 6370 viral-host protein pairs for direct interaction (Supplementary Tables 5 and 6), of which 587 correspond to proximal proteins identified by BioID (Supplementary Table 6).

Among the tested viral-host protein pairs, regardless of their status in BioID, 6% (414/6372) showed direct interactions involving 118 host proteins. In the set of protein pairs detected by BioID, 21% (123/587) were direct, indicating a 3.5-fold enrichment (Supplementary Table 6). To benchmark the quality of our mN2H dataset, we compared the detection rates between our exploratory BioID dataset and the previously established human positive and random (negative) reference sets (PRS and RRS)^31^. At a stringent threshold of 0% RRS (i.e. 0% false negative), the % of direct PPI found within proximal virus/host pairs from the BioID dataset was similar to the direct PPIs recovery in the PRS datasets (Fig. S2, Supplementary Table 7). Therefore, the SARS-CoV-2-host direct interactions detected by mN2H show a quality equivalent to highly studied and curated direct interactions. In contrast, no significant difference was observed between the randomly explored PPI interactions, regardless of their status in BioID, and the RRS dataset (Supplementary Fig. S2). The mN2H SARS-CoV-2-host contactome determined in this study is provided in Fig. 3b, uniquely identifying 151 direct interactions involving 41 host factors essential for SARS-CoV-2 lethality in 3 + CRISPR-Cas9 screens.

**Fig. 4:**
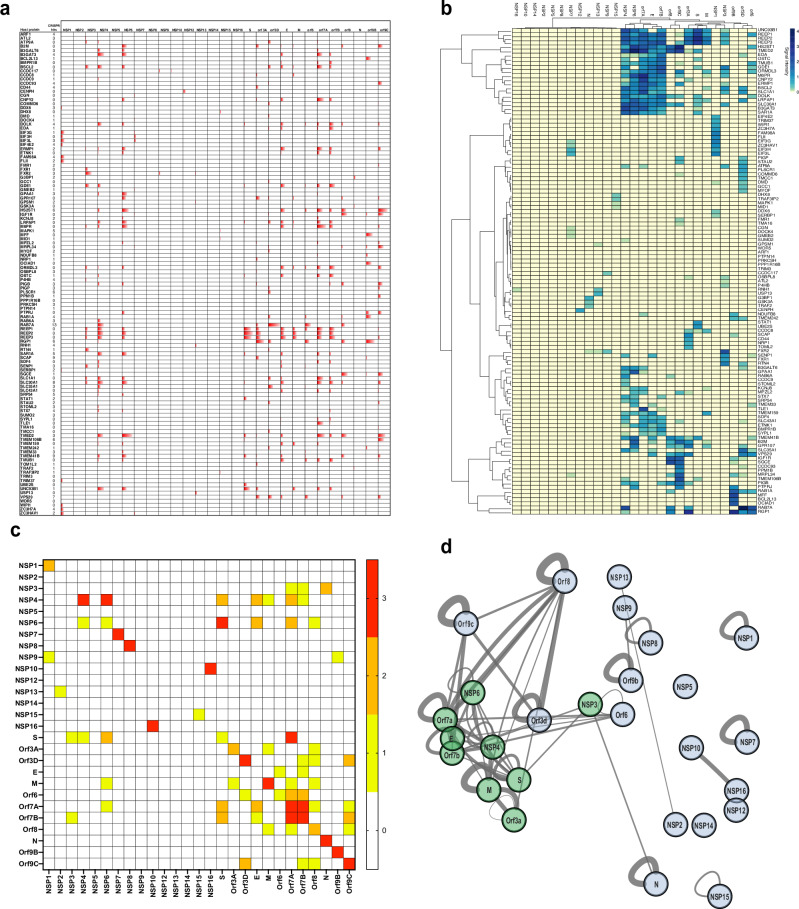
mN2H interaction profiling. **a** The relative strength of PPIs, given by the distance of PPI to the positive threshold, is shown for host factors scoring positive with one or more viral factors. Their corresponding number of CRISPR hits is indicated (*n* = 4 biologically independent samples). **b** Hierarchical clustering of the SARS-CoV-2 proteins and human host proteins based on their mN2H interaction profiles. The heatmap represents relative PPI strength (distance to the threshold) (*n* = 6 biologically independent samples). **c** Heatmap of intraviral interactions tested in mN2H, interactions were tested in triplicate using three different complementary NanoLuc configurations. The color gives the number of N2H tagging configurations where interactions scored positive (*n* = 2 samples per configuration). **d** Network representation of intraviral interactions. The thickness of the lines represents the number of configurations (N1-N2, N1-C2, C1-C2) scoring positive in N2H. The viral proteins colored in green represent the proteins harboring a transmembrane domain, while the proteins colored gray represents the ones without a transmembrane domain.

Signal intensities detected in split-nanoluciferase assays correlate with the interaction strength^32^, allowing comparison of interactions according to their distance from the positive PPIs threshold. Such a comparative intensity map (Fig. 4a) informs both on the redundancy of host factor targeting and the prominent host targets per viral factor, both characteristics strongly increasing mapping accuracy. The hierarchical clustering of mN2H PPI profiles of SARS-CoV-2 proteins essentially reflects the 3D map (Fig. 4b), supporting the idea that direct contacts shape the virus-host spatial organization. PPI profile-based clustering separates viral proteins into two main groups: the first one consists of most of the non-structural proteins that each exhibit few contacts with host proteins, while the second cluster consists of organelles-associated viral proteins that engage in the majority of direct interactions. The most striking host cluster corresponds to proteins involved in ER organization, which is strongly co-opted by a subset (but not all) of viral membrane proteins (NSP4, NSP6, E, ORF7a, ORF7b). Comparing the interaction profiles discriminates these viral proteins, with NPS4/NSP6 clustering together and E, ORF7a and 7b on the other side (Fig. 4b). This is an example of the precision offered by mN2H PPIs profiling to highlight the intricate and finely tuned modulation of direct interactions essential for the functions of viral proteins and their orchestration along the viral cycle. Other host clusters appear specific to a given viral protein, such as proteins involved in translation initiation (4 members of the EIF family) or in phosphatidylinositol processes (FLII and WIPI1) that are especially associated with NSP1, while mitochondrial proteins are specifically contacting ORF9b (BCL2L13, MFF, MRPL34, NDUFB8 and TMEM242).

### Intraviral interaction profiling substantiates viral proteins’ spatial positioning

In the course of the virus life cycle, viral proteins cooperate to orchestrate the reprogramming of the cell machinery. As viral proteins interacting together are usually localized within the same region of the cell, we sought to map virus-virus PPIs by mN2H and compare the results with the clusters of proteins localized together in our 3D map. Several known intra-virus binary PPIs were detected, such as between N and NSP3^33^ or the NSP10-NSP16 dimer^34^ (Fig. 4d, Supplementary Table 8). In addition, a hub of intra-viral PPIs emerges within the set of organelles-associated viral factors lying close together in the 3D network and the hierarchical clusters (NSP3, NSP4 and NSP6, E, S, M, ORF7a and ORF7b) (Fig. 4d). The intraviral PPI data thus corroborate the accuracy of the positioning of the viral proteins in the 3D map.

### Predictive value of the BioID-based 3D interaction map

**Fig. 5:**
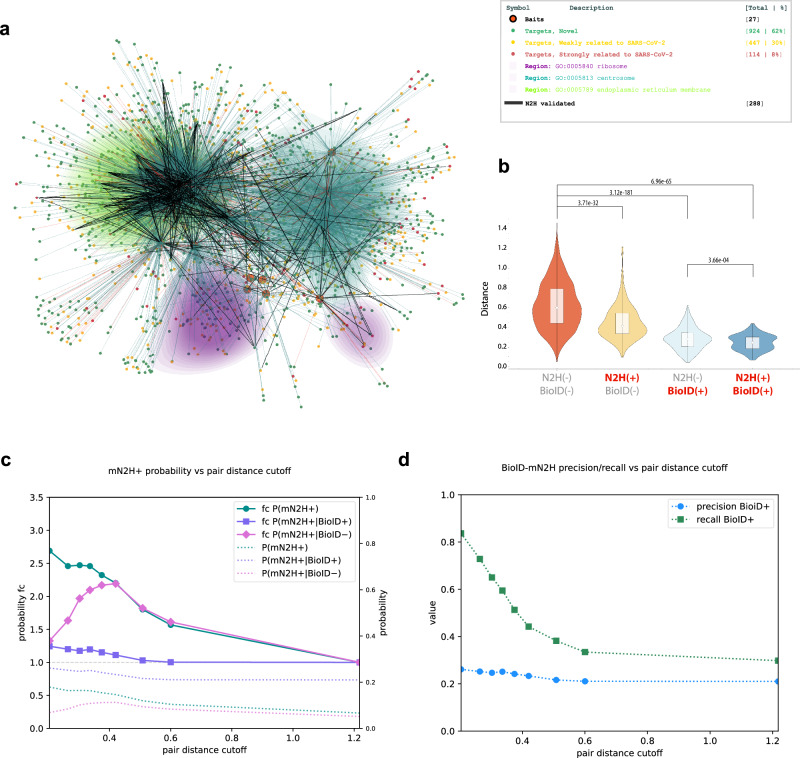
Organization and validation of the BioID and mN2H networks. **a** Snapshot of the 3D network depicting spatially resolved and colored regions enriched in a subset of GO categories. Merged data from the present study and the literature are graphically coded as described in the legend. **b** Violin plot of the viral-host distances in the 3D map according to the PPIs status, as described (Student’s *t* test; *p*-values as indicated in the figure). **c** Dependence of the probability of mN2H positive interactions on protein pair distance cutoff (conditioned on BioID detection status), depicted as absolute numbers and as fold change relative to total (max cutoff), for bins of approximately equal mN2H+ pair populations. **d** Dependence of BioID positive status precision and recall on protein pair distance cutoff for predicting mN2H positive status, for bins of approximately equal mN2H+ pair populations.

Since proteins localizing close to each other in a cell have higher chances of directly interacting together, we sought to assess if our 3D map could have a predictive value in identifying potential direct interactions. We leveraged the data obtained by mN2H to determine the predictive strength of our 3D map. Remarkably, we observed a striking spatial confinement of mN2H-positive host proteins around their viral direct partners, as discerned from the BioID-based 3D map (Fig. 5a). We note that mN2H information was not used at any stage of the 3D layout of the interactome graph, thus maintaining the orthogonality of the two methods and preventing distortion due to the partial virus-host PPI coverage by mN2H. We examined the distances between the viral proteins and host factors based on four categories: (***i***) non-interacting, non-proximal proteins (PPIs not detected by either method); (***ii***) proximal proteins (PPIs detected by BioID only) (***iii***) direct-contact proteins within the BioID dataset (PPIs detected by both BioID and mN2H) (**iv**) *de-novo* identified direct-contact proteins (PPIs detected by mN2H only). We report that directly interacting proteins detected by mN2H and identified within the 6372 viral-host protein pairs tested in mN2H were separated by shorter distances on average than non-interacting proteins (based on mN2H negative signal). This remained true when restricting to the subsets of proteins detected by BioID, as well as not detected by BioID: mN2H-positive interactions showed an average distance lower than mN2H-negative ones in both cases (Fig. 5b, Supplementary Fig. S3A–C). While the mN2H-tested matrix showed an average viral-host protein distance of 0.58 (in normalized map coordinates), pairs identified by mN2H-only, BioID-only and mN2H&BioID were at average distances of 0.45, 0.29 and 0.25, respectively (Fig. 5b). Additionally, we calculated the probability of interactions to be positive based on their distance to the bait on the 3D map. We show that interactions with closer distances between the bait and the prey on the 3D map have a higher probability of being positive in mN2H (Fig. 5c). We also calculated the probability fold change to identify the distances between bait and prey at which the probability of being positive in mN2H changes the most. Interestingly, the probability fold change for interactions negative in BioID and positive in mN2H is 2 at distances of 0.3 and increases to 2.1 at distances of 0.5 before falling rapidly at higher distances, suggesting that interactions with distances between 0.3 and 0.5 have higher chances of being positive in mN2H (Fig. 5c). It is noteworthy that at distances shorter than 0.3, most direct PPIs interactions have also been detected by BioID, and almost no de novo detection of direct PPIs by N2H are observed (Fig. 5c).

Calculating the precision and recall of mN2H positive interactions among the BioID proximal partners based on the distances between bait and prey, we found that at distances shorter than 0.2, our recall is 90% with a precision of 20%, meaning that among 20% of the proximal partners identified in BioID, 90% are positives in mN2H (Fig. 5d). Moreover, we found that when moving to larger distances, both the precision and recall decreased, supporting the idea that our 3D map can predict direct interactions based on distances.

These results strongly bolster the predictive power of the 3D network layout in foretelling volumes enriched in direct PPIs, underscoring the efficacy of our approach in capturing key interactions critical for viral-host dynamics.

### Modeling of virus-host complexes interaction interfaces

As PPIs play an essential role in regulating biological functions and the development of diseases such as COVID-19, targeting their interaction interface for disruption is an interesting strategy. Using the *in-silico* prediction tool AlphaFold-Multimer^35^, we assessed the power of our mN2H matrix of direct interactions to provide virus-host interaction interfaces. On a selection of 70 virus-host PPIs scoring positive by mN2H, a predicted structure of acceptable confidence was provided for 10 (pDockQ >0.23)^36^ or about 14% of them (Supplementary Fig. S4, Supplementary Table 9). Both the number of predicted structures and the pDockQ values are unsurprisingly lower than the ones previously reported for human datasets^36^ as most of the viral proteins are known to be challenging to predict due to a lack of heterologs in the AlphaFold database^37^.

**Fig. 6:**
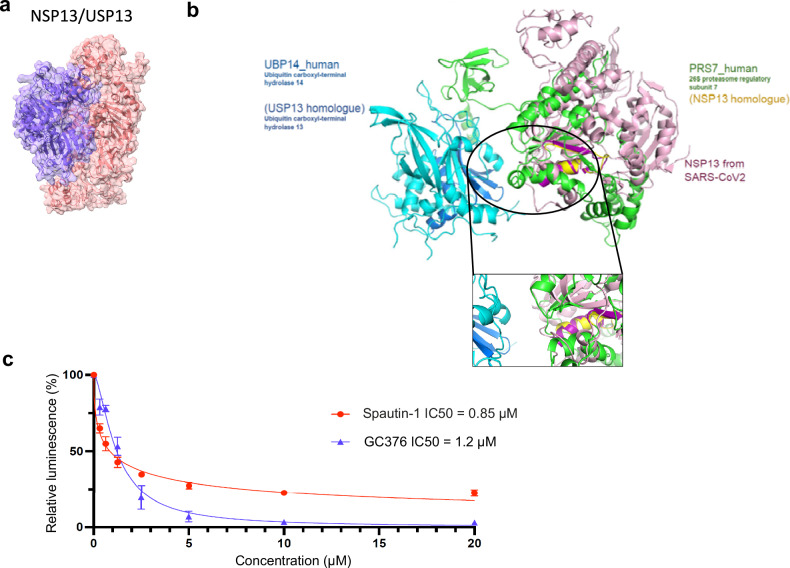
Structural modeling and validation of the NSP13-USP13 role in SARS-CoV-2 replication. **a** Modeling of the USP13-NSP13 complex by Alphafold-Multimer and homology-based prediction (**b**). **c** Inhibition curve of SARS-CoV-2 infection in the presence of Spautin-1, using the nanoluciferase complementation assay. Briefly, a co-culture of Vero E6-NanoLg and Vero E6-NanoSm, plated at equal density the day before infection, was infected at a MOI of 0.01 with the Wuhan strain of SARS-CoV-2. Increasing concentrations of Spautin-1 (red) and GC376 (blue) were added at the time of infection. Luciferase was measured 24 h post-infection as a read-out of infection-induced syncytia formation (*n* = 4 biologically independent samples).

The interaction with the highest pDockQ score (0.433) was the NSP13-USP13 interaction (Fig. 6a), involving the NSP13 viral helicase and the human deubiquitinase USP13^38^. This complex was also obtained by homology-based structural modeling with the human UBP14-PRS7 complex (Fig. 6b), providing a robust interaction interface.

Having successfully modeled structures for several PPIs furnishes a potent dataset to dissect their role in SARS-CoV-2 infection and pave the way of anti-viral drug discovery.

### Targeting human components of the modeled virus/host complexes to identify potential therapeutics

To estimate the relevance of targeting virus/host complex with PPIs-disruptive compounds, we need to ensure that the cellular protein plays a role in infection in SARS-CoV-2, which is particularly required when the protein partner was not detected in CRISPR screen or other functional studies, as for USP13. We thus developed a novel assay, called Nano-Fuse (Supplementary Fig S5A) to measure SARS-CoV-2 infection through nanoluciferase activity, instead of the classical time consuming and low-throughput plaque forming assays. In this assay, a co-culture VeroE6 cell clones stably expressing, on one hand a Large nanoluciferase-derived fragment deleted from its 13 last residues, and on the another hand the 13 aa C-terminal peptide of Nanoluciferase. Upon co-culture, SARS-CoV-2-induced cell fusion leads to the reconstitution of an active luciferase whose activity correlates with the infectious viral titer (Supplementary Fig S5B). The specificity and sensitivity of the assay have been thoroughly validated (Fig. 5b), and we used it to test the effect of the Spautin-1, a previously reported USP13 inhibitor^38^. We observed a dose-dependent decrease in SARS-CoV-2 infection upon Spautin-1 treatment (Fig. 6c). The protease inhibitor GC376, assessed in the same assay, had a 50% inhibitory concentration (IC50) of 1.2 µM, which ascertains that the nanoluciferase complementation assay properly reflects an active SARS-CoV-2 infection (Fig. 6c). Spautin-1 inhibited SARS-CoV-2 infection with a calculated IC50 of ~0.8 µM more efficiently than GC376 while no toxicity of the compound was observed (Fig. 6c). The dose-response profile suggests that USP13 is important but non-essential for SARS-CoV-2 replication since the inhibition plateaued at 80%, which aligns with its lack of detection in the CRISPR screens. Nevertheless, our findings conclusively validate USP13’s role in SARS-CoV-2 infection, thus deserving further screening of PPI disruptive compounds.

## Discussion

Since the beginning of protein interaction mapping, one goal has been to map and localize the interactions inside the cell. However, this process has been challenging due to the lack of detection methods able to capture distances between proteins and the limited application of network layouts in 3D. Our study presents a pipeline to generate a 3D map modeled from proximal interaction data (BioID). Applying this pipeline to SARS-CoV-2, we modeled the spatial representation of the SARS-CoV-2/human interactome within a cell-like volume, combining our own BioID SARS-CoV-2 dataset and a set of already identified host-host interactions. These latter allow a better approximation of the coordinates for each of the viral and host proteins in the 3D map. While large interaction networks generally lack accuracy, this methodology improves the accuracy of large interaction networks generally making them more accurate due to the availability of data to finely tune the positioning of nodes.

Several improvements can still be built upon the foundation of this work. Currently, the work is constructed only using the SARS-CoV-2 BioID dataset, with proteins being represented within a cube, referred to as an abstract cell volume. Machine learning algorithms could be used to draw an actual cell volume and transfer the current 3D map layout within this new volume based on known protein location. The recently developed OpenCell database, providing a catalog of protein subcellular localization^39^, will likely be helpful to achieve a more accurate representation of the viral-host interactome in the real cellular architecture.

Additionally, the produced 3D map, similar to every other interaction network, is static, while interactions and protein localization are known to be dynamic. By performing proximity-dependent biotinylation experiments with shorter labeling time, such as TurboID allowing for time points every 10 or 30 min, and using machine learning, a dynamic interaction network of SARS-CoV-2 inside the cell could be modeled. Moreover, the integration of other studies will be beneficial for incrementally refining protein coordinates. Despite these limitations, our pipeline takes us one step closer to a more reliable and biologically relevant representation of interactomes, which can be applied to complex PPI networks. Applied to the SARS-CoV-2 virus-host PPI network, this representation provides key insights by depicting the invasion pattern of cell territories by the viral proteins and by reflecting virus coordination to coopt the cell machinery along the viral cycle.

Furthermore, using an orthogonal screening of direct virus-host contacts by mN2H, we demonstrated the predictive value of such an approach in identifying direct PPIs. Host factors located at shorter distances are enriched in direct partners of viral proteins and can be prioritized for N2H profiling, increasing the recovery rate of direct host contact identification. Mapping of intraviral direct interactions by mN2H identified hubs of direct contacts between viral proteins that were also clustered in the 3D map, showing the accuracy of our pipeline to position interacting proteins at shorter distances.

A number of virus/host complexes that we identified have been validated and functionally characterized recently, such as Spike/TMEM106B reported as essential for an ACE-2 independent SARS-CoV-2 entry^21^, N/G3BP1 involved in viral replication^22^, NSP3/FXR1 mediating stress granule disruption^23^ and ORF3A/Rab7A impacting on lysosome function^24^. These data highlight the functional significance of intra-virus and virus/host PPIs discovery and characterization for a better understanding of the viral life cycle. Overlaying our interactomics data with data from CRISPR screens provides potent entry points for drug discovery and deep biological understanding of pathogenesis.

In silico structural modeling of SARS-CoV-2-host complexes provided interaction interfaces, which can be leveraged for structure-directed molecule screening. The human USP13 de-ubiquitinase was modeled binding to the NSP13 viral helicase both by structure-based homology and AlphaFold-Multimer^35^, and we demonstrated the involvement of USP13 in SARS-CoV-2 replication, substantiating the functional significance of the virus/host PPI resource generated.

Additionally, the host-viral protein complexes predicted with AlphaFold can indicate which protein regions are prone to be involved in PPI interfaces, thus providing insights into potential epitopes for targeting and disrupting these PPIs. PPIs binding affinities can also be investigated from AlphaFold models using platforms such as PDBePISA. Moreover, the combination of BioID and mN2H allows the detection of direct PPIs in a semi-quantitative manner, wherein the intensity of the luminescence signal correlates with the interaction intensity^32^, thereby offering additional indications about protein binding affinities.

This PPI discovery pipeline is implementable in a record time just upon viral genome sequencing, independently of any in-vitro culture system, which makes it ideally suited to improving preparedness against emerging viruses.

## Methods

### Plasmids

SARS-CoV-2 viral proteins coding sequences were cloned by Gateway recombination system (Life Technologies) into pDEST pcDNA5 FRT/TO N-ter and C-ter BirA*Flag vectors for the BioID (Coyaud et al.^40^), and into pciNeo-N2 or pciNeo-C2^31^ using the collection of Gateway-compatible entry clones reported in Kim et al.^41^ (see Supplementary Table 1 for sequences). For N2H, cDNA encoding the human proteins to be tested were available pDONR vectors from the human ORFeome collection v8.1 from the Center for Cancer Systems Biology (CCSB) (ORFeome Collaboration, 2016) or inhouse made clones.

Each hORF was introduced into pciNeo-N1 pDEST plasmids by Gateway reaction. All clones were sequence verified.

### Cell lines

HEK293 cells were stably transfected by the different BirA*tag- encoding plasmids as in (Coyaud et al.^40^). Briefly, Flp-In™ T-REx™ HEK293 cells were grown in Dulbecco’s Modified Eagle’s Medium (DMEM, Gibco) supplemented with 10% fetal bovine serum (FBS, Sigma-Aldrich), GlutaMAX™ and Penicillin-Streptomycin (1x). Using the Flp-In system (Invitrogen), Flp-In™ T-REx™ HEK293 stably expressing BirA*Flag or FlagBirA* alone (for control samples), or N- and C-terminally tagged viral bait proteins were generated by co-transfecting pOG44 with each pcDNA5 FRT/TO BirA*Flag-viral protein encoding plasmid and selected with 200 μg/ml hygromycin B.

### BioID sample generation

BioID samples were prepared from Flp-In™ T-REx™ HEK293 expressing each BirA*-flagged SARS-CoV-2 protein or BirA*alone as in Coyaud et al.^40^. Briefly, three independent replicates of two 150 cm^2^ plates of sub-confluent (60%) cells were incubated for 24 h in complete media supplemented with 1 μg/ml tetracycline (Sigma), 50 μM biotin (Thermo Fisher Scientific). Cells were collected and pelleted (300 × *g*, 3 min), washed twice with PBS, and dried pellets were snap frozen. Each cell pellet was resuspended in 5 ml of lysis buffer (50 mM Tris-HCl pH 7.5, 150 mM NaCl, 1 mM EDTA, 1 mM EGTA, 1% Triton X-100, 0.1% SDS, 1:500 protease inhibitor cocktail (Sigma-Aldrich), 1:1,000 Turbonuclease (BPS Bioscience) and incubated on an end-over-end rotator at 4 °C for 1 h, briefly sonicated to disrupt any visible aggregates, then centrifuged at 45,000 × *g* for 30 min at 4 °C. Supernatant was transferred to a fresh 15 mL conical tube. 25 μl of packed, pre-equilibrated Streptavidin Ultralink Resin (Pierce) were added, and the mixture incubated for 3 h at 4 °C with rotation. Beads were pelleted by centrifugation at 300 × *g* for 2 min and transferred with 1 mL of lysis buffer to a fresh eppendORF tube. Beads were washed once with 1 mL of lysis buffer and twice with 1 mL of 50 mM ammonium bicarbonate (pH = 8.3), then transferred in ammonium bicarbonate to a fresh centrifuge tube and washed two more times with 1 ml of ammonium bicarbonate buffer. Tryptic digestion was performed by incubating the beads with 1 μg MS-grade TPCK trypsin (Promega, Madison, WI) dissolved in 200 μl of 50 mM ammonium bicarbonate (pH 8.3) overnight at 37 °C. The following morning, 0.5 μg MS-grade TPCK trypsin was added to the beads and incubated 2 additional hours at 37 °C. Following centrifugation at 2000 × *g* for 2 min, the supernatant was collected and transferred to a fresh eppendORF tube. Two additional washes were performed with 150 μL of 50 mM ammonium bicarbonate and pooled with the first eluate. The sample was lyophilized and resuspended in buffer A (2% ACN 0.1% formic acid). 1/3rd of each sample was analyzed per mass spectrometer run.

### BioID data acquisition

MS samples were prepared from three biological replicates of each bait protein fused either with an N-terminal or a C-terminal BirA*Flag epitope tag in basal condition prior to tetracycline and biotin induction, and analyzed on a Thermo Q-Exactive mass spectrometer. Samples were separated by online reversed-phase chromatography using a Thermo Scientific Easy-nLC1000 system equipped with a Proxeon trap column (75 μm ID × 2 cm, 3 μm, Thermo Scientific) and a C18 packed-tip column (Acclaim PepMap, 75 μm ID × 50 cm, 2 μm, Thermo Scientific). The digested peptides were separated using an increasing amount of acetonitrile in 0.1% formic acid from 2 to 30% for 2 hours at a 300 nL/min flow rate. A voltage of 2.4 kV was applied by the liquid junction to electrospray the eluent using the nanospray source. A high-resolution mass spectrometer Q-Exactive™ Thermo Scientific™ was coupled to the chromatography system to acquire the 10 most intense ions of MS1 analysis (Top 10) in data-dependent mode. The MS analyses were performed in positive mode at a resolving power of 70,000 FWHM, using an automatic gain control target of 3e6, the default charge state was set at 2 and a maximum injection time at 120 ms. For full scan MS, the scan range was set between m/z 300 to 1600. For ddMS2, the scan range was between m/z 200 to 2000, 1 microscan was acquired at 17,500 FWHM, an AGC was set at 5e4 ions, and an isolation window of m/z 4,0 was used. The mass spectrometry proteomics data have been deposited to the ProteomeXchange Consortium via the PRIDE^42^ partner repository with the dataset identifier PXD033452.

### BioID data analysis

The proteins were identified by comparing all MS/MS data with the Homo sapiens proteome database (Uniprot, release March 2020, Canonical+Isoforms, comprising 42,360 entries +viral bait protein sequences added manually), using the MaxQuant software version 1.5.8.3. The digestion parameters were defined using trypsin with 2 maximum missed cleavages. The oxidation of methionine and N-terminal protein acetylation were defined as variable modifications. The Label-free quantification (LFQ) was done keeping the software’s default parameters. As for initial mass tolerance, 6 ppm was selected for MS mode, and 20 ppm was set for fragmentation data to match MS/MS tolerance. The identification parameters of the proteins and peptides were performed with a false discovery rate (FDR) at 1%, and a minimum of 2 unique peptides per protein. The LFQ values from the 30 control runs (regrouping FlagBirA* and BirA*Flag alone samples, from stable and transiently transfected cell lines, were collapsed to the three highest values for each given ID. These three values were defined as the control group for comparison with viral bait proteins triplicates. The statistical analysis was done by Perseus software (version 1.6.2.3). Briefly, the LFQ intensity of each sample was downloaded in Perseus, and the data matrix was filtered by removing the potential contaminants, reverse and only identified by site. The data were then transformed using the log2(x) function. Before statistical analysis, 81 groups (27 bait proteins, N-ter and C-ter each) were defined with 3 replicates per group. Only preys with detected values in all three replicates of a given viral bait protein were kept for further analysis. Missing values were then replaced from the normal distribution separately for each column. Two-sample Student’s *T* test was then performed comparing all three biological replicates of each bait and condition against the three control runs. High-confidence proximal interactors were defined by permutation-based FDR with a cut-off of 0.01. Perseus output with all experimental values is reported in Supplementary Table 2 (tab B).

The matrix (Supplementary Table 2) shows the average log2 fold change against control and the corresponding *p*- and *q*-values for each bait and condition. The InDegrees column depicts the number of bait proteins detecting a given interactor, regardless of the condition (N-ter or C-ter). This criterion was chosen to filter out the most connected interactors (8+), likely to be organelle-specific background not filtered using the BirA* alone control samples. 2D networks presented in the different figures were generated using the Cytoscape software (v.3.9.1; https://cytoscape.org/).

### Spatial modeling of the virus-host interplay from the BioID Data set

In order to approximate the regions where proteins mainly reside in the cell volume, we employed well-established computational methods for the layout of graphs in three dimensions. A custom force-directed and multi-stage algorithm was used due to its general applicability and capacity for the generation of high-quality layouts^43^. Another advantage offered by the class of force-directed algorithms, making it suitable for our specific case, are the inherent physical attributes and the flexibility of tuning provided by the underlying physics-inspired model producing the layout coordinates. In this approach, the observed BioID proximal interactions (when present) modulate the strength of an attractive force between proximal protein pairs, while repulsive forces (universal among all node pairs) ensure the separation of regions and the occupation of the available volume. In this way, host proteins previously known to interact are pulled close to each other and viral proteins with overlapping sets of host interactors are also brought into relative proximity. Interactors for which there is no pairwise proximal relation are pushed apart, their placement determined by their own set of individual proximal interactions. By simultaneously aggregating all the interactors and their interactions subject to these forces, a global approximate picture of the relative spatial organization of viral proteins with respect to the host proteome is emerging.

### Interactome network setup and visualization

The starting point for constructing the interactome network is the BioID high confidence PPI data table, each row of the table containing data of a single bait-target (or viral-host protein) interaction. Aggregating all the PPI data, we obtained a total of 10,125 interactions involving 27 baits and 2593 targets (total of 2620 nodes). Based on this dataset, we construct a weighted undirected graph where interactors and interactions are represented by the graph’s vertices (nodes) and edges (links) respectively. The edge weights correspond to the strength of interaction as determined by BioID. In addition to links due to the observed BioID bait-target interactions, we augment the graph with 3492 edges (of equal weight) corresponding to known target-target interactions, extracted from the IID orphid database (https://iid.ophid.utoronto.ca/), as experimentally confirmed in 2+ independent studies. We thus obtain a denser graph with a total of 2620 vertices and 13,617 edges. In this graph we finally append 290 mN2H positive interactions not detected by BioID – these latter PPIs are not participating in the layout and therefore do not influence the node coordinates, but are used for displaying purposes.

To visualize the graph, our objective is to determine an optimum placement of the nodes in three-dimensional space that would minimize edge crossings and reveal the interactome’s structure to the best possible extent. We employ a custom, multi-stage, force-directed graph layout algorithm in 3D, based largely on the spring-electric model outlined by Hu^43^. We modulate the strength of the attractive (spring) forces in the corresponding physical model with a factor *w* given by the combination of the BioID enrichment ratio (or fold change, *fc*) according to the formula: *w* = [log_2_ (fc)_i_] *p*, where *p* = ½ and *i* = {N-ter, C-ter}, when present for the interaction. For reported target-target interactions where this information is not available we set this factor equal to 1. We also ignore the previously mentioned purely mN2H-positive (non-BioID) interactions in order to preserve the orthogonality of the two approaches. The node coordinates in 3D obtained by minimizing the energy of the spring-electric physical model are ingested by a plotting package that generates a 3D visualization of the network, assigning a point to each node and a line connecting a pair of nodes when there is an interaction. Our code was written in Python 3.10^44^ and makes use of several modules, primarily: NetworkX for graph operations^45^, NumPy for numerical computations^46^, pandas for data manipulation^47^ and Plotly for visualization^48^.

### Interactive web application

In order to make our results available to the wider community in a usable and informative manner, we have implemented a web application accessible at http://dev.sars-cov-2-interactome.org/. It contains network visualization, as well as multiple options for selecting and filtering the dataset to view sub-networks, providing the ability to focus on particular areas of interest and explore various levels of detail. The interactive 3D visualization of the interactome is a main ingredient of the dashboard, as it enables navigation within a browser window, zooming and rotating in space, giving an intuitive illustration of the relative localizations of proteins. In addition, we integrate information about the previously reported SARS-CoV-2 status of interactors and interactions, as well as previously reported interactions between the host proteins (cf. Supplementary Table 2 for details). The network customization options contain various filters, separated broadly into two levels: on the first level, there are options for: (i) focusing on targets of higher specificity by filtering out targets with a total number of bait interactions (or degree) above a configurable threshold (set to 7 by default); (ii) restricting to targets based on known relevance for SARS-CoV-2 (based on total number of CRISPR screens an interactor has been identified as functionally important for SARS-CoV-2 infection); or (iii), selecting particular baits of interest and their interactions only. When multiple filters are activated at this level, the resulting dataset is formed by the intersection of the selected criteria. In addition, at this level, we provide options for displaying the previously reported target-target interactions, as well as highlighting previously reported and N2H-validated bait-target interactions. At the second level, there is the functionality to select any number of annotations of interest from three main categories (pathways, biological processes, cellular components) in order to generate an annotated subnetwork, while remaining subject to the filtering selections from the preceding level. In this second level, multiple annotation selections are combined so that the resulting network is the union of the individually annotated subnetworks. Specific targets of interest can also be selected individually, or added to an already generated subnetwork. Notably, there is an option to select any number of annotations for which to display their approximate localization overlaid on the network, either as 3D density volumes, or as labels positioned at the volume’s centroids (forming a ‘label cloud’). At any given time, the interactive visualization reflects the user’s filtering selections, offering an intuitive view of the particular subset of data under consideration, while the filtered dataset is also visible in table format below the network and also downloadable. Finally, there are options for mostly cosmetic attributes of the network visualization, such as the ability to toggle text labels and edges, present a faded view of the full network in the background (thus giving a better global view of the location of nodes and edges in the interactome), and display 3D spherical shapes for the interactors (as opposed to flat 2D circular markers), better representing their physical size and actual location in space. A summary panel of the network with a breakdown of the totals for nodes and edges is presented alongside the network, as well as a panel displayed upon clicking on a node or edge with corresponding metadata, including AlphaFold protein structure predictions. A detailed usage tutorial is also provided as part of the website. The dashboard web application has been implemented using the Dash module in Python 3.10.

### Mammalian cell-based N2H assay

HEK293T cells were seeded at 6 × 10^4^ cells per well in 96-well, flat-bottom, cell culture microplates, and cultured in Dulbeccoʼs modified Eagleʼs medium (DMEM) supplemented with 10% fetal calf serum at 37 °C and 5% CO_2_. Twenty-four hours later, cells were transfected with 100 ng of each N1- and N2/C2-expressing plasmids using linear polyethylenimine (PEI MAX 40000; Polysciences Inc; Cat# 24765) to co-express the protein pairs fused with complementary NanoLuc fragments, F1 and F2^31^. Twenty-four hours after transfection, the culture medium was removed, and 30 μL of 100× diluted NanoLuc substrate^49^ was added to each well of a 96-well microplate containing the transfected cells. Luciferase enzymatic activity was measured using a CentroXS luminometer (Berthold; 2 s integration time). The luciferin substrate used (Q-108) in all the bioluminescence experiments was obtained in a concentrated solution, from the corresponding O-acetylated derivative hikarazine-108, following acidic hydrolysis as previously described^50^. The binary virus-host PPIs were assessed and were independently performed two times, each made in duplicates to quadruplicates. PPIs were monitored using the cellular proteins (CP) fused the F1 nanoluciferase fragment at their N-terminus (N1-CP), while the viral proteins were fused to the nanoluciferase F2 fused either at N-, their C-terminus or both. Configuration of the virus-host PPIs matrix was either N1N2 or N1C2, and was selected from the viral bait Bir-A* fusion configuration leading to host partners identification in BioID.

To score positive PPIs from the tested matrixes, we applied the following pipeline:

1-Log2-transformed averaged RLU signals were normalized to the mean columns signal and mean row signals (Log2 RLU PPI - mean Log2 column - mean Log2 rows). Column and row averages reflect the overall level of viral and cellular protein interaction signals. A Z-score (relative to the mean and standard deviation of the full Normalized PPIs matrix) was applied on normalized data to obtain normal distribution and to calculate a *P*-value. A significant *P*-value (<0.05) or equivalently a Z-score upper to 1.64 were scored as positive PPIs. In this way, positive PPIs are distinguished over non-interacting pairs with high stringency, leading to the identification of high-confidence PPIs.

Hierarchical clustering of virus-host PPIs was performed using the R software by using euclidean distance parameter of the dist function. Clustering was then performed by ward.D method with R software by using the pheatmap function.

### AlphaFold-Multimer in silico structure prediction

We produced predicted complex structures for the selected pairs of viral-host proteins by running the AlphaFold source code v2.3.2 (git commit hash 2e6a78f) from https://github.com/deepmind/alphafold in multimer mode with default parameters and with full databases via the provided Docker script. For each pair, we ran using input host protein sequence FASTA files from https://www.uniprot.org/ and generated 5 relaxed structures (from different random seeds) for each of the 5 multimer models. We kept the top-ranked relaxed structure in terms of model confidence in order to compute a predicted DockQ (pDockQ) score^51^. Following the CAPRI definitions for the correspondence between DockQ values and accuracy of predicted structures, we deem structures with pDockQ ≥0.23 as positive hits for the purposes of our in silico validation.

### A novel sensitive assay to measure SARS-CoV-2 infection

cDNA encoding for the nanoluciferase fragment 1–158 (NanoLg) containing the same mutations than the LargeBiT fragment of the Nanobit assay^52^ and the 13 aa long c-terminal Nanoluciferase peptide (aa 159–171, Nano-Sm) were cloned in the pLVX-puro vector (Clontech) in fusion with GFP11 (at the C-ter of LN) or GFP1-10 (at the N-ter of SN) respectively. Lentiviral vectors were produced by co-transfection of the pLVX-puro NanoLg or pLVX-puro NanoSm plasmids with gag-pol packaging plasmid psPAX2 (Addgene, #12260) and VSV-G envelope expressing plasmid in HEK293T cells using calcium phosphate precipitation. Lentiviral titers were measured by p24 ELISA according to the manufacturer’s instructions (Clontech).

10^5^ cells Vero E6 were transduced with 5.10^6^ TU NanoLg or NanoSm expressing lentiviruses for 4 h, then cells were grown in DMEM 5% SVF + 1% Penicillin-Streptomycin +8 µg/ml puromycin. Transduced cells were then amplified under puromycin treatment.

For the SARS-CoV-2 infection assay, Vero E6-NanoLg and Vero E6-NanoSm were plated separately at 25,000 cells/well or co-cultured (12,500 of each cell lines mixed/well) in white 96 well plates (Greiner Bio-One, #655083). One day after, cells were infected by serial dilutions of SARS-CoV-2 strain 1973 (hCoV-19/France/GES-1973/2020, EPI_ISL_414631) in 50 µl of DMEM, then incubated for 24 h. Infection rates were measured by lysing cells after removal of the medium in 30 µl of 100-fold diluted NanoLuc substrate (Promega, #N1110). Luciferase enzymatic activity was measured using a CentroXS luminometer (Berthold; 2 s integration time).

### Small molecule inhibition

GC376 (Sigma Aldric) and Spautin-1 (Calbiochem) were dissolved in DMSO to make a 100 mM stock solution. A 40 mM solution was prepared from the stock in DMSO, and then serial 2-fold dilutions were prepared in DMSO. The final range of inhibitors was from 40 mM to 0.63 mM (i.e. 8 2-fold dilutions), which is 200 times higher than the tested concentrations. Each compound dilution was diluted 1000-fold in DMEM (giving 2X concentrated chemicals with equal DMSO concentration for each).

Vero E6 NanoLg + Vero E6 NanoSm co-cultures (12,500 each) plated the day before in 96 white plates were infected with 500 PFU (MOI 0.01) of the previously described SARS-CoV-2 strain in 50 µl DMEM for 1 h. Then 50 µl of the 2X concentrated compounds were added. Cells were incubated for 24 h. Infection rate was measured by nanoluciferase activity reading as described above.

Each compound was measured in biological triplicates, each containing 3–8 technical replicates.

The results are presented as RLU ± standard deviations. Dose-response curves were plotted using Prism v9 (GraphPad) in which IC_50_ values were calculated with the variable slope model.

Cell viability was measured using the Cell-Titer-Glo^®^ Luminescent Cell Viability Assay kit (Promega, G7750).

### Statistics and reproducibility

We performed all statistical analyses using either R-Studio, Python or Prism (GraphPad Software, version 8). The figure legends specify the number of replicates and the type of tests conducted. The numbers of biological replicates were chosen based on the nature of the experiments and published papers describing similar experiments. To compare two groups, we used a two-tailed unpaired Student’s *t* test. Fisher’s exact test was performed on 2 × 2 contingency to determine if the difference of proportion of positive direct interactions was significant between random pairs tested in mN2H and pairs tested in mN2H and coming from our BioID results. Data in graphs are presented as mean ± S.E.M. Data distribution was assumed to be normal but this was not formally tested. The study sizes were not predetermined using statistical method. The experiments were not randomized, and investigators were not blinded to allocation during experiments.

### Reporting summary

Further information on research design is available in the Nature Portfolio Reporting Summary linked to this article.

## Supplementary information

Supplementary information

Reporting Summary

## Data Availability

The mass spectrometry proteomics data have been deposited to the ProteomeXchange Consortium via the PRIDE^42^ partner repository with the dataset identifier PXD033452. Supplementary tables are available on Figshare (10.6084/m9.figshare.28515860). The source data are available on Figshare (10.6084/m9.figshare.28528268).

## Code availability

The code used in this paper is available upon request.
