# Supplementary material for: Multimodal SARS-CoV-2 interactome sketches the virus-host spatial organization
Source: Commun Biol. 2025 Mar 26;8:501. doi: 10.1038/s42003-025-07933-z (PMC11947133; doi:10.1038/s42003-025-07933-z)
Supplement: Supplementary file 1 — Supplementary information [file 42003_2025_7933_MOESM1_ESM.pdf]

Supplementary information

Multimodal SARS-CoV-2 interactome sketches the virus-host spatial organization

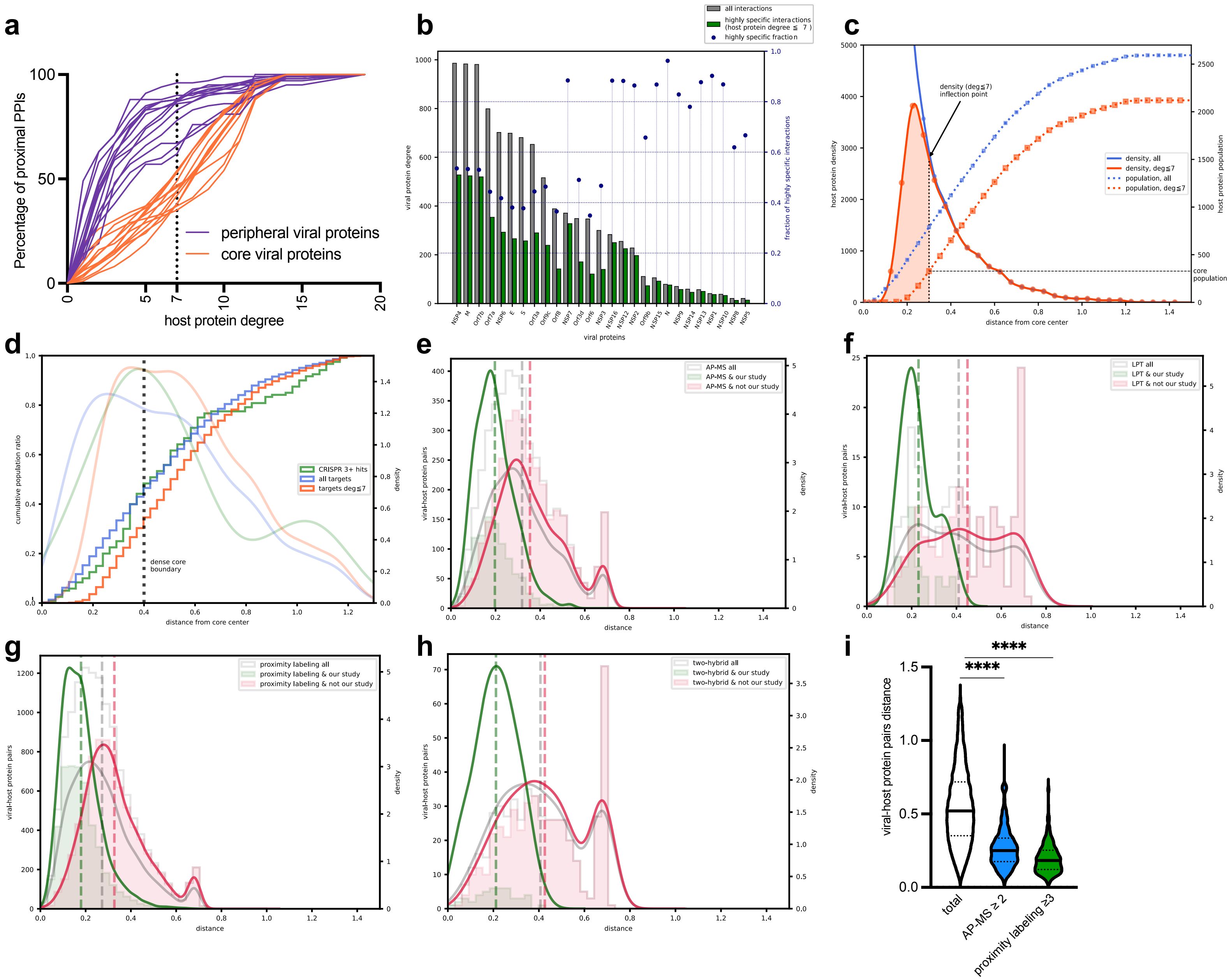

Fig S1. Quantitative analysis and features of the BioID-based network and 3D map. Analysis of the effect of filtering out host proteins with BioID degree greater than seven on the network topology: (**a**) Percentage of proximal PPIs for core viral proteins and peripheral viral proteins based on host protein degree. (**b**) Histogram of individual viral protein degree before and after filtering for highly specific interactors. (**c**) Host target density and total population dependence with radial distance from core center for the full and pruned (host protein degree <= 7) networks. Estimation of dense core size by the radial value of the inflection point in the density interpolated line for the case of the pruned network. Host proteins below the cutoff are considered to belong inside the dense core. (**d**) Population fraction of host proteins, host proteins with BioID degree <= 7 and host proteins detected in 3+ CRISPR studies residing within a radial distance from the dense core center. (**e**) Histogram of 3D map distances for virus-host protein interactions detected by AP-MS studies; totals and breakdown by BioID detection status in this study. (**f**) Histogram of 3D map distances for virus-host protein interactions detected by low throughput studies; totals and breakdown by BioID detection status in this study. (**g**) Histogram of 3D map distances for virus-host protein interactions detected by proximity labeling studies; totals and breakdown by BioID detection status in this study. (**h**) Histogram of 3D map distances for virus-host protein interactions detected by two-hybrid studies; totals and breakdown by BioID detection status in this study. (**i**) Violin plot of the distances for the total virus-host protein interactions, the interactions identified in more than two AP-MS studies and the interactions identified in more than three proximity labeling studies.

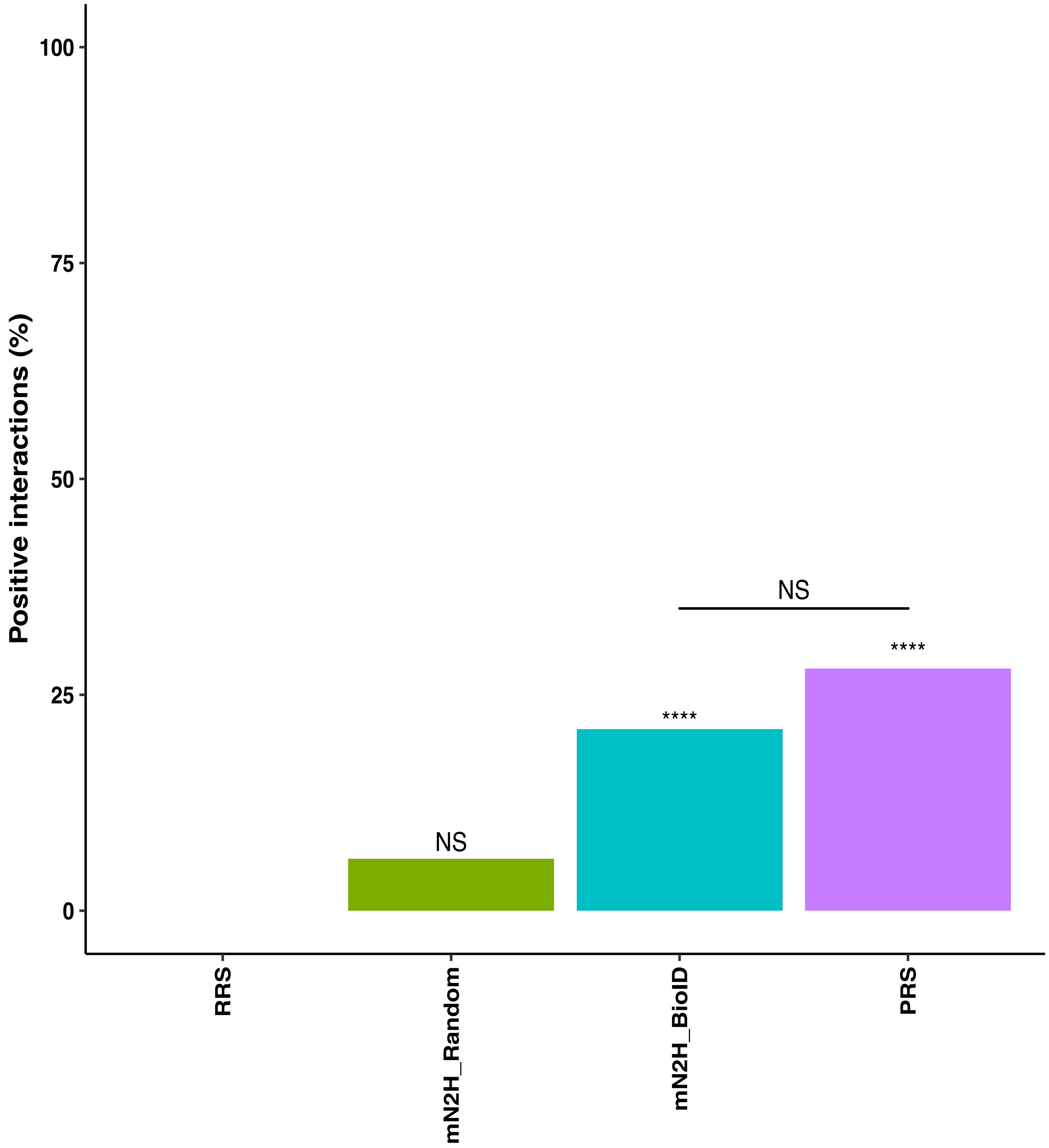

Fig S2. Comparison of the positive interaction percentages in different mN2H datasets. Percentage of positive interactions in the positive control (PRS), negative control (RRS), BioID derived mN2H (BioID_mN2H) and BioID random (BioID_random) sets. Asteriks represent a significant difference from the RRS benchmarking set ("****" : P-value < 0.05, NS : non significant, Fischer's exact test with FDR correction).

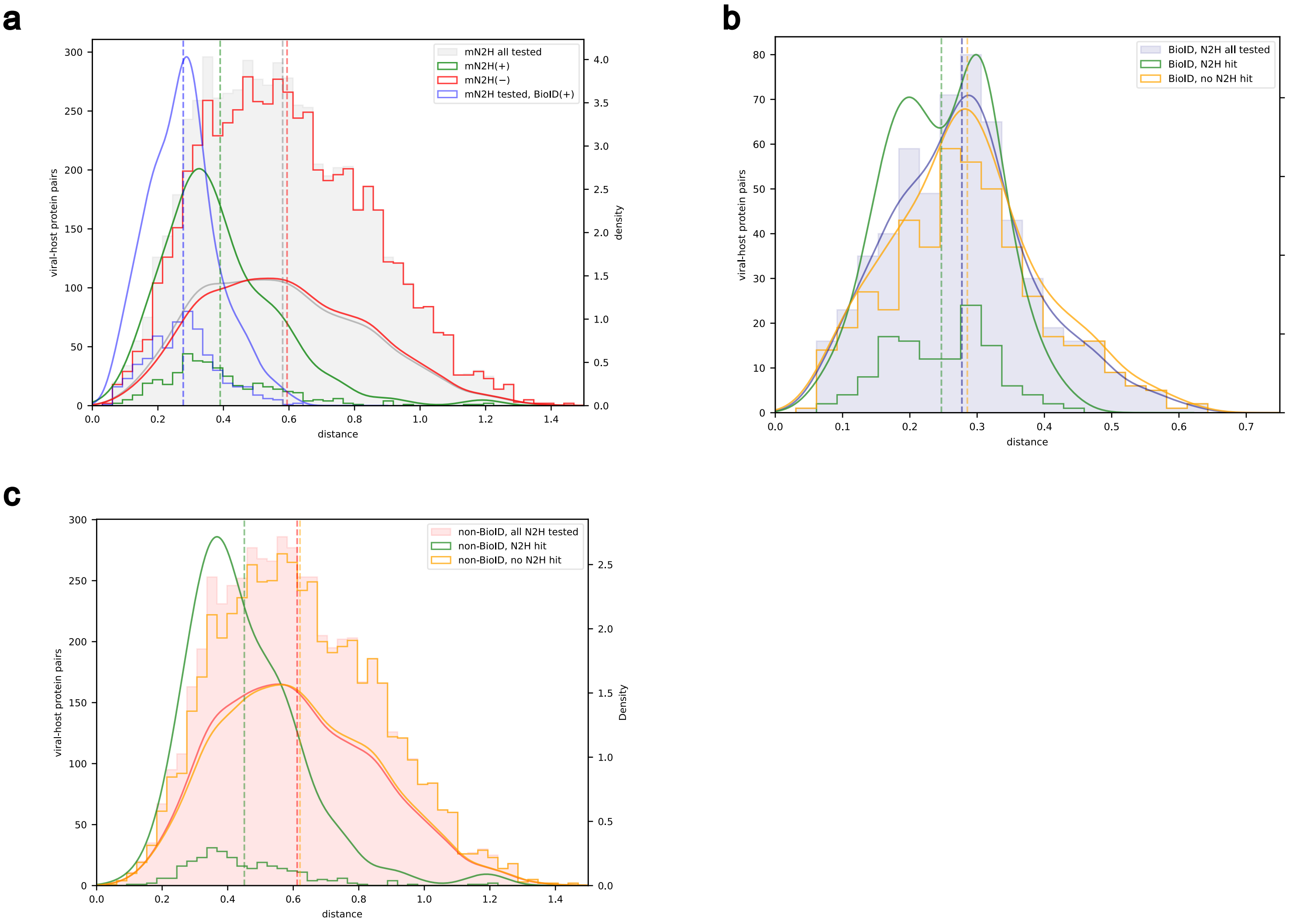

Fig S3. Pairwise distance analysis for BioID and mN2H orthogonal methods. (**a**) Distance histograms, densities and averages (vertical dashed lines) for all viral-host protein pairs tested in mN2H, including breakdown by mN2H +/- status, and subset of BioID+ interactions. mN2H+ positive pairs are found at lower average distances, their distribution deviating significantly from the overall mN2H matrix distribution and showing similarity to the BioID+ distribution. (**b**) Distance histograms, densities and averages (vertical dashed lines) restricted to mN2H-tested BioID+ protein pairs including breakdown by mN2H+/- status. Despite having no influence on the construction of the 3D layout, mN2H+ pairs exhibit a lower average distance, reinforcing the predictive power of the map. (**c**) Distance histograms, densities and averages (dashed lines) restricted to mN2H-tested BioID- protein pairs and breakdown by mN2H+/- status. Despite having no influence on the construction of the 3D layout, N2H+ pairs exhibit a lower average distance, reinforcing the predictive power of the map.

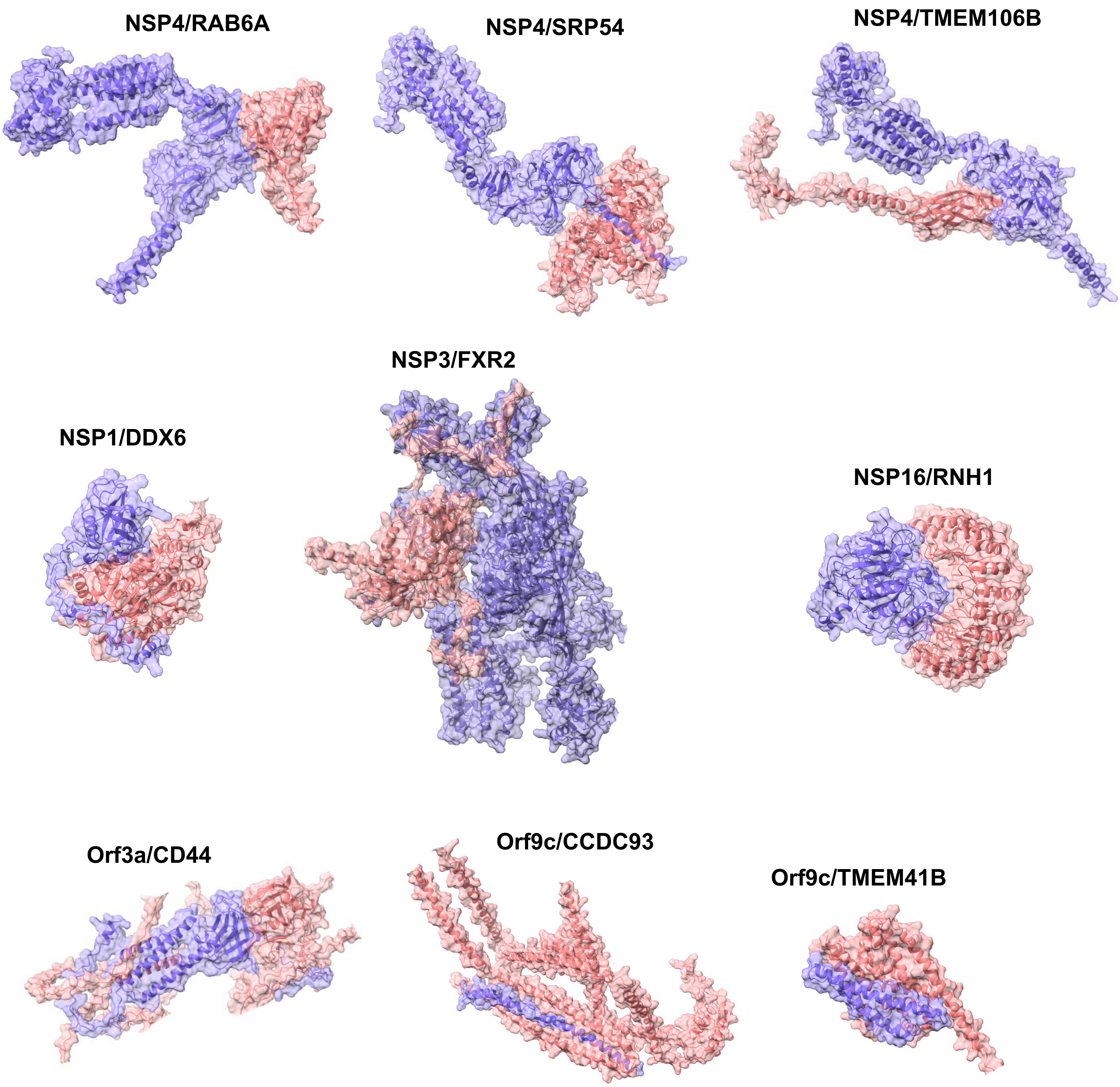

Fig S4. AlphaFold-Multimer predicted structures of SARS-CoV-2-host complexes. Cartoon representation of the SARS-CoV-2-Human protein complexes with a pDockQ score >0.23. The viral proteins are shown in light violet, and the host proteins are in light pink. Protein surfaces are shown. When no structure prediction was available, the resulting unstructured representations obtained by AlphaFold were not shown for the corresponding proteins, owing to the lack of information they bring. The *.pdb files corresponding to each predicted complex are provided as hypertext links in **Dataset EV9**.

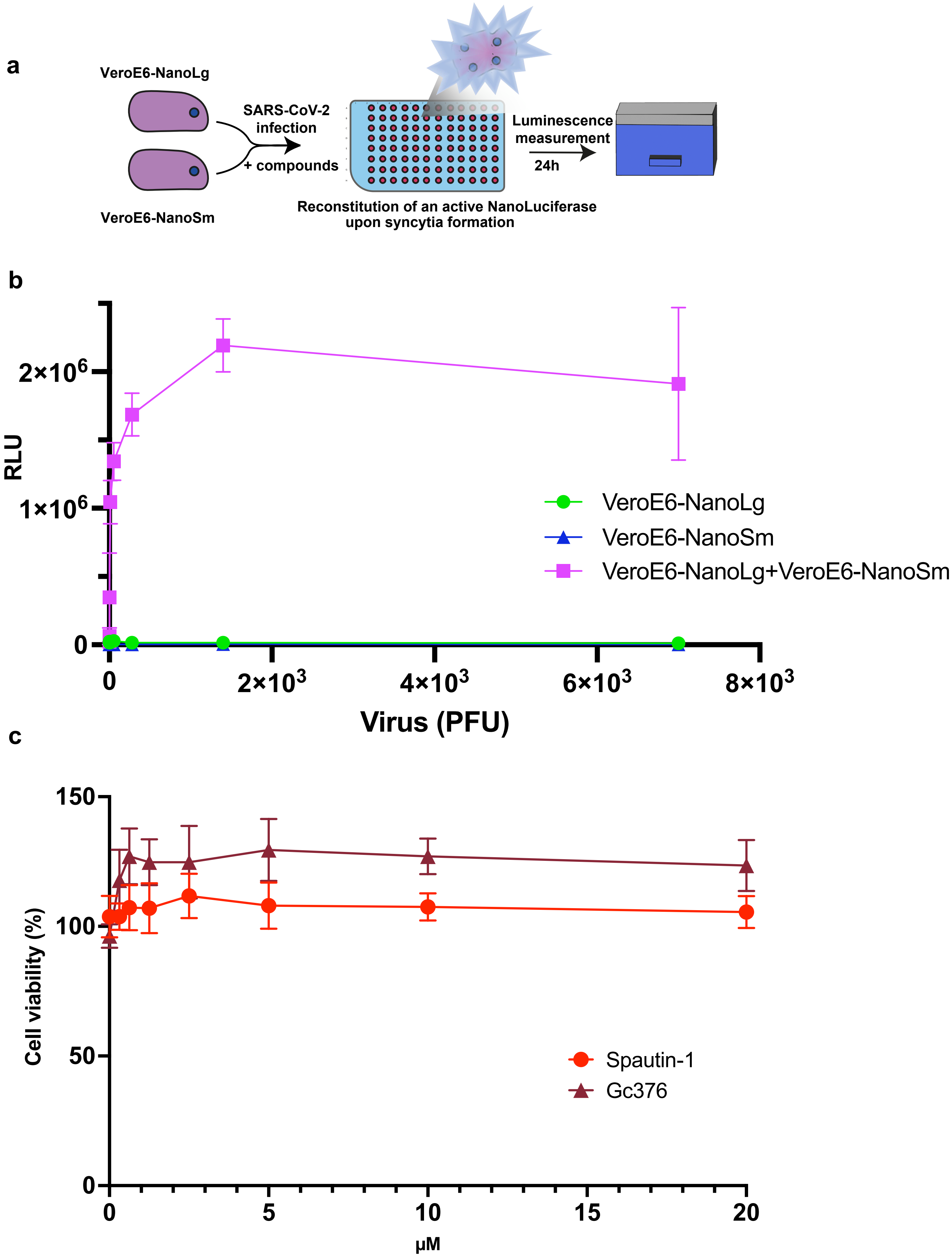

Fig S5. Benchmarking of the Nanoluciferase auto-complementation system for SARS-CoV-2 infection. (**a**) Illustration of the NanoLuciferase auto-complementation system. (**b**) Vero E6-NanoLg and Vero E6-NanoSm, either cultured separately or co-cultured, equal density were infected with serial dilutions of the Wuhan strain of SARS-CoV-2. Luciferase was measured 24 hours post-infection as a read-out of infection-induced syncytia formation. (**c**) Cell viability assay performed on cells treated with the indicated amounts of Spautin-1 or Gc376, and compared with DMSO-treated cells.

Supplementary tables 1-9 are available on Figshare (10.6084/m9.figshare.28515860)

Supp table 1. List and sequences of SARS-CoV-2$_{wuhan}$ ORFs used in this study. Each gene synthesize cDNA encoding viral proteins from the early SARS-CoV-2 strain WuhanHu-1 (GenBank MN908947[54]) where obtained from Kim et al.,2020[40] as gateway pENTRY clones and transferred in the BioID or mN2H-dedicated plasmids.

Supp table 2. SARS-CoV-2 BioID high-confidence data. Tab A. Full high-confidence proximal interactors. The number of baits detecting a given prey protein is indicated in the 'Indegree' column. The 'Abundance vs background' column represents the ratio between the detected prey in given bait assays against the control group (see Tab for RawQuant Perseus data). For each cellular protein, the number of CRISPR screens where they scored as necessary for SARS-CoV-2 infection is indicated. Tab B. Raw output of the Perseus analysis of MaxQuant LFQ results. Tab C. ToppCluster analysis for enriched/ gained interactors. Tab D. Reported SARS-CoV-2-host interactions. Tab E. Reported interactions between the host factors. Tab F. Overview of the hit numbers according to configuration. Tab G. Number of PPIs per ORFs before and after filtering at 7 indegrees. Tab H. Metadata of the BioID experiments. Tab I. Comparison of the PPIs identified in our study against the Hoffmann et al, Li et al and Sheng et al studies.

Supp table 3. Factors of the dense core (less than 0.3 of the centroid in the 3D map coordinates). Tab A. List of factors in the dense core. Tab B. GO enrichment analysis of factors.

Supp table 4. List of the virus-host protein distances in the 3D map. List of the virus-host distances in the 3D map, and of their status in each PPI detection method.

Supp table 5. mN2H raw data and normalization. Mean RLU of quadruplicates (Fold Change or FC matrices) or duplicates to quadruplicates (CRISPR matrices) of mN2H PPIs are given along with the normalization pipeline for mN2H positive signals scoring

Supp table 6. Summary of the mN2H data. The distance to the positive PPIs threshold is given for each virus-host PPIs tested. PPIs detection in previous proximal interactomics and physical interactomics studies is given, as well as the number of CRISPR Hits of the host factor.

Supp table 7. mN2H raw data and normalization for the PRS-RRS datasets. Raw data and analysis for the PRS-RRS datasets.

Supp table 8. Intra-virus mN2H raw data and normalization. Tab A. Raw data and analysis for the N1N2 configuration. Tab B. Raw data and analysis for the C1N2 configuration. Tab C. Raw data and analysis for the C1C2 configuration. Tab D. Summary of the intraviral results for each of the configurations tested.

Supp table 9. Predictions of SARS-CoV-2-host complexes. Tab A. pDockQ scores AlphaFold-Multimer structures, interactions represented in **Fig. S4** are highlighted in red. Tab B. Confidence scores (ipTM+pTM scores) of AlphaFold-Multimer structures. pdb

files of the corresponding predicted virus-host complex structures are accessible through hypertext links.
